# Lymphatic Malformation, Retinoblastoma, or Facial Cleft: Atypical Presentations of PHACE Syndrome

**DOI:** 10.1155/2015/487562

**Published:** 2015-06-28

**Authors:** María Fernández-Ibieta, Juan Carlos López-Gutiérrez

**Affiliations:** ^1^Pediatric Surgery Service, Hospital CU Virgen de la Arrixaca, El Palmar s/n, 30150 Murcia, Spain; ^2^Vascular Anomalies Unit, Pediatric Surgery Department, Hospital La Paz, Madrid, Spain

## Abstract

PHACE syndrome is a neurocutaneous disorder characterized by large cervicofacial infantile hemangiomas and associated anomalies: posterior fossa brain malformation, hemangioma, arterial cerebrovascular anomalies, coarctation of the aorta and cardiac defects, and eye/endocrine abnormalities of the brain. When ventral developmental defects (sternal clefting or supraumbilical raphe) are present the condition is termed PHACE. In this report, we describe three PHACE cases that presented unique features (affecting one of the organ systems described for this syndrome) that have not been described previously. In the first case, a definitive PHACE association, the patient presented with an ipsilateral mesenteric lymphatic malformation, at the age of 14 years. In the second case, an anomaly of the posterior segment of the eye, not mentioned before in PHACE literature, a retinoblastoma, has been described. Specific chemotherapy avoided enucleation. And, in the third case, the child presented with an unusual midline frontal bone cleft, corresponding to Tessier 14 cleft. Two patients' hemangiomas responded well to propranolol therapy. The first one was followed and treated in the pre-propranolol era and had a moderate response to corticoids and interferon.

## 1. Introduction

PHACE association affects 2.3% of all patients with infantile hemangioma (IH) and consists of a plaque-like IH in a “segmental” dermatomal distribution of the face with at least one of the following anomalies: posterior fossa brain malformation, hemangioma, arterial cerebrovascular anomalies, coarctation of the aorta and cardiac defects, and eye/endocrine abnormalities. When ventral developmental defects (sternal clefting or supraumbilical raphe) are present the condition is termed PHACES [1–3]. Cerebrovascular anomalies are the most common associated finding (72%) [1, 4]. Less than one-third of children have more than one extracutaneous feature. Because 8% of patients might have a stroke in infancy and 42% have a structural brain anomaly, patients with suspected PHACE association should have an MRI to evaluate brain structures and vasculature. Treatment is multidisciplinary and the angiomatous plaque usually responds well to propranolol [1–3].

## 2. Cases Presentation

### 2.1. Case 1

**Figure 1:**
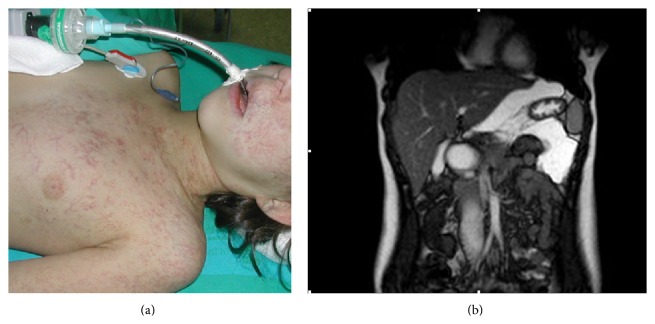
Left angiomatous cervicofacial and thoracic hemangioma plaque and lymphatic mesenteric malformation that surrounded stomach and spleen inferior pole.

A 14-year-old girl with a history of PHACES association consisting in left angiomatous cervicofacial and thoracic plaque (Figure 1), left cerebellar hypoplasia, and moderate hypoplasia of ipsilateral internal carotid and ophthalmic artery presented with 6-month history of abdominal pain and distention. She had been treated with corticoids and interferon *ά*2B for the hemangioma in the pre-propranolol era (with acceptable evolution, but she had neurologic sequelae consisting in fine movements incoordination). On abdominal examination, moderate ascites was found. In ultrasound, a well-defined liquid mass or cyst appeared, and a thoracoabdominal RM was performed. The final diagnose was a lymphatic mesenteric malformation, which surrounded stomach and spleen inferior pole, with a left caudal extension that compressed medially the bowel (Figure 1). A laparoscopy was performed, and the lymphatic malformation was enucleated from the mesentery, without recurrence.

### 2.2. Case 2

**Figure 2:**
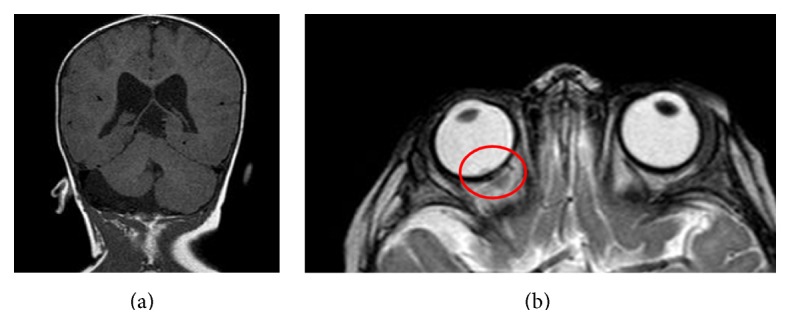
Right cerebellar and vermis hypoplasia plus a 2 mm length tumor of the retina (retinoblastoma).

An 8-month-old male infant with well-defined PHACE association (right facial hemangiomatous plaque with right cerebellar and vermis hypoplasia plus a posterior fossa arachnoid cyst and pituitary cystic malformation, Figure 2) with good response to propranolol therapy presented with strabismus of the right eye. On ophthalmoscopic examination, a retinal papula was found. An ultrasound showed a 2 mm length tumor of the retina, and an eye RM was performed, where retinoblastoma was defined (Figure 2). Chemotherapy was then established, and enucleation was spared, with no relapse after one year follow-up.

### 2.3. Case 3

**Figure 3:**
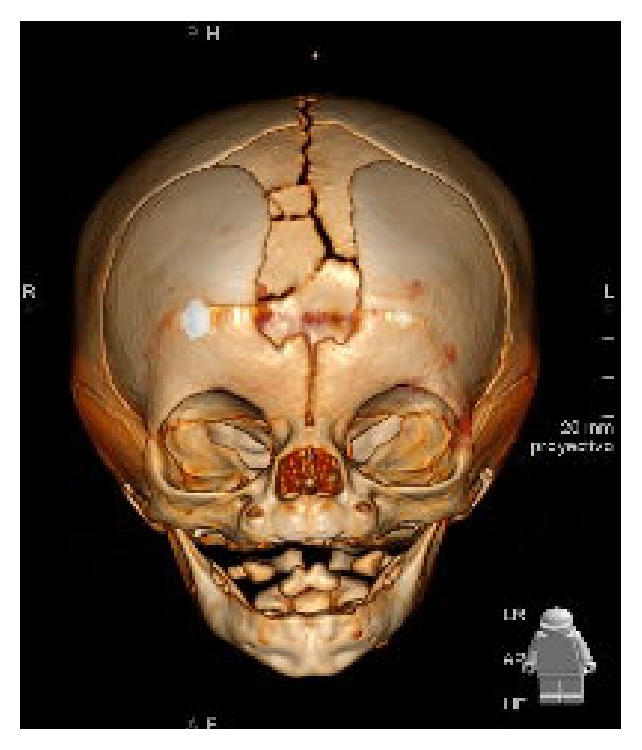
Craniofacial cleft, corresponding to a Tessier 14 midline craniofacial cleft, between nose and facial bone.

A one-month-old female presented in our office with facial and cranial angiomas. A cranial midline soft tissue protuberance was also observed. Magnetic resonance imaging of the brain showed a craniofacial cleft, corresponding to a Tessier 14 midline craniofacial cleft, between nose and facial bone (Figure 3). There were no intracranial lesions. A thyroglossal cyst was also found on the tongue base. The angiomatous plaques responded well to propranolol.

In this report, we describe three PHACE cases that are associated with unique features (affecting one of the organ systems described for this syndrome) that have not been described previously.

## 3. Discussion

The association between facial IH and cerebral anomalies was first described in 1978, and, since then, PHACES association has been recognized as the most frequent phacomatosis or neurocutaneous anomaly [5]. Its differential diagnosis comprises, among others, the Sturge-Weber syndrome, which is defined as capillary malformation (instead of a hemangioma) that is already present at birth, opposite to IH, which develops along the first weeks of life [5]. The hemangiomas in PHACES tend to be big (>5 cm) and evolve as a usual hemangioma in three phases, including development, arrest, and involution. Plus, it is seen in approximately 30% of those with >5 cm segmental hemangioma patients. They are segmental plaque-like hemangiomas, derived from embryological protuberances known as placodes, which contain neural crest cell migrated from the dorsal crest and develop into the mandibular, maxillar, and frontonasal processes [6, 7]. This may explain why patients with posterior fossa malformations have arterial and cutaneous anomalies in the same dermatome. A consensus statement on diagnostic criteria for PHACE syndrome has been reached recently [8], and the anomalies have been described as major or minor criteria. The most frequent structural brain anomalies are Dandy-Walker-type malformation and ipsilateral cerebellar hypoplasia. Cerebrovascular anomalies include hypoplasia or tortuosity of major vessels (internal carotid, anterior, middle or posterior cerebral arteries, or vertebrobasilar system) and persistence of embryonic vessels. These usually affect neurological development and most severe anomalies may present with stroke, global developmental delay, or seizures. The most cardiovascular malformations are coarctation of aorta, right-sided aortic arch, and ventricular septal defect. These are usually associated with sternal/umbilical/midline defects. Ophthalmological anomalies include posterior segment abnormalities (optic nerve hypoplasia and persistent fetal vasculature). Moreover, hypopituitarism and thyroid anomalies have been described [8–12]. According to the above-mentioned consensus, PHACE syndrome is defined in the presence of a plaque-like facial hemangioma and a major criterion (these are most prevalent features as posterior fossa anomalies, dysplasia of the large cerebral arteries, aortic arch anomaly, eye posterior segment anomaly, or sternal defect) or two minor criteria (callosal agenesis, pituitary malformation, ventricular septal defect, eye anterior segment anomaly, hypopituitarism, or ectopic thyroid, among others). The consensus defined the term “possible PHACE syndrome” when the patient presented with hemangioma/hemangiomatous plaque plus 1 minor criteria.

We have reported three PHACE cases, with unique features that have not been described before. In the first case, a definitive PHACE association, the patient presented with an ipsilateral mesenteric lymphatic malformation, at the age of 14 years. This finding might be more frequent as its clinical presentation is often symptomless for years until the cyst develops and causes pain or intestinal symptoms. The second case, again a definitive PHACE syndrome, an anomaly of the posterior segment of the eye, not mentioned before in PHACE literature, a retinoblastoma, has been described. And, in the third case, possible PHACE syndrome (facial hemangioma with an ectopic thyroid) presented with an unusual midline frontal bone cleft, corresponding to Tessier 14 cleft [13]. It is reasonable to speculate that if thoracic hemangiomas may associate midline sternal/umbilical clefts, facial ones could present cranial midline defects. Two patients' hemangiomas responded well to propranolol therapy [14–17]. The first one was followed and treated in the pre-propranolol era and had a moderate response to corticoids and interferon.

## References

[1] Greene A. K. (2011). Management of hemangiomas and other vascular tumors. *Clinics in Plastic Surgery*.

[2] Smithers C. J., Fishman S. J. (2010). Vascular anomalies. *Ashcraft's Pediatric Surgery*.

[3] Kulungowski A. M., Fishman S. J. (2012). Vascular anomalies in Coran-Grosfeld's. *Pediatric Surgery*.

[4] Haggstrom A. N., Garzon M. C., Baselga E. (2010). Risk for PHACE syndrome in infants with large facial hemangiomas. *Pediatrics*.

[5] Metry D., Heyer G., Hess C. (2009). Consensus statement on diagnostic criteria for PHACE syndrome. *Pediatrics*.

[6] Waner M., North P. E., Scherer K. A., Frieden I. J., Waner A., Mihm M. C. (2003). The nonrandom distribution of facial hemangiomas. *Archives of Dermatology*.

[7] Castillo M. (2008). PHACES syndrome: from the brain to the face via the neural crest cells. *American Journal of Neuroradiology*.

[8] Oza V. S., Wang E., Berenstein A. (2008). PHACES association: a neuroradiologic review of 17 patients. *American Journal of Neuroradiology*.

[9] Metry D. W., Dowd C. F., Barkovich A. J., Frieden I. J. (2001). The many faces of PHACE syndrome. *Journal of Pediatrics*.

[10] Ruiz-de-Luzuriaga A. M., Bardo D., Stein S. L. (2006). PHACES association. *Journal of the American Academy of Dermatology*.

[11] Heyer G. L., Millar W. S., Ghatan S., Garzon M. C. (2006). The neurologic aspects of PHACE: case report and review of the literature. *Pediatric Neurology*.

[12] Heyer G. L., Dowling M. M., Licht D. J. (2008). The cerebral vasculopathy of PHACES syndrome. *Stroke*.

[13] Bradley J. P., Kawamoto H. (2007). *Craneofacial Clefts and Hypertelorbitism in Grabbs and Smith's Plastic Surgery*.

[14] Drolet B. A., Frommelt P. C., Chamlin S. L. (2013). Initiation and use of propranolol for infantile hemangioma: report of a consensus conference. *Pediatrics*.

[15] Bronzetti G., Patrizi A., Giacomini F. (2014). A PHACES syndrome unmasked by propranolol interruption in a tetralogy of fallot patient: case report and extensive review on new indications of *β* blockers. *Current Medicinal Chemistry*.

[16] Sharma V. K., Fraulin F. O. G., Dumestre D. O., Walker L., Harrop A. R. (2013). Beta-blockers for the treatment of problematic hemangiomas. *Canadian Journal of Plastic Surgery*.

[17] Lynch M., Lenane P., O'Donnell B. F. (2014). Propranolol for the treatment of infantile haemangiomas: our experience with 44 patients. *Clinical and Experimental Dermatology*.

